# Analyses of balance and flexibility of obese patients undergoing bariatric surgery

**DOI:** 10.6061/clinics/2016(02)05

**Published:** 2016-02

**Authors:** Fernanda Antico Benetti, Ivan Leo Bacha, Arthur Belarmino Garrido Junior, Júlia Maria D'Andréa Greve

**Affiliations:** I Faculdade de Medicina da Universidade de São Paulo, Departamento de Ortopedia e Traumatologia, Laboratório do Estudo do Movimento, São Paulo/, SP, Brazil; II Faculdade de Medicina da Universidade de São Paulo, Departamento de Cirurgia do Aparelho Digestivo, São Paulo/, SP, Brazil

**Keywords:** Obesity, Morbid Obesity, Bariatric Operation, Postural Balance, Flexibility

## Abstract

**OBJECTIVE:**

To assess the postural control and flexibility of obese subjects before and both six and 12 months after bariatric surgery. To verify whether postural control is related to flexibility following weight reductions resulting from bariatric surgery.

**METHODS:**

The sample consisted of 16 subjects who had undergone bariatric surgery. All assessments were performed before and six and 12 months after bariatric surgery. Postural balance was assessed using an *Accusuway^®^* portable force platform, and flexibility was assessed using a standard chair sit and reach test (Wells' chair).

**RESULTS:**

With the force platform, no differences were observed in the displacement area or velocity from the center of pressure in the mediolateral and anteroposterior directions. The displacement speed from the center of pressure was decreased at the six month after the surgery; however, unchanged from baseline at 12 months post-surgery. Flexibility increased over time according to the three measurements tested.

**CONCLUSIONS:**

Static postural balance did not change. The velocity of postural adjustment responses were increased at six months after surgery. Therefore, weight loss promotes increased flexibility. Yet, improvements in flexibility are not related to improvements in balance.

## INTRODUCTION

Obesity is a chronic disease characterized by the excessive accumulation of adipose tissue in the organism 1,2 and the prevalence of obesity has increased markedly during the last decades 3. Francischi et al. 4 and Bankoff et al. 5 report that obesity is an epidemic that affects both developed and developing countries and constitutes a worldwide health problem 4,5.

In the obese subject, the maintenance of postural balance and body stability are more difficult during walking and position changes, although the support basis provided by the foot position is proportional to the structural morphology of each subject. Other factors also interfere with postural balance maintenance, including body mass distribution; height of gravity center; anthropometric relationships between anatomical structures (trunk/thorax/abdomen/pelvis/lower limbs; and foot position. Their greater inertia makes it more difficult for obese people to maintain stability and postural balance 6.

People with good flexibility move easier and tend to suffer fewer problems related to pain and muscle and joint lesions, especially in the lumbar region 7. Flexibility is gradually impaired in obese subjects and these changes can be related to postural changes, which are worsened by a sedentary lifestyle and biological aging. These factors further worsen the motor performance of obese subjects 8.

Bariatric operations are indicated for patients with a body mass index (BMI) >40 or BMI >35 associated with sleep apnea, type II diabetes mellitus, arterial hypertension, dyslipidemia, locomotion difficulties and other areas that are difficult to manage clinically 9,10,11. The objectives of bariatric operations are to reduce comorbidities and improve the quality of life for the patient 12. In addition to weight loss in patients who have undergone bariatric operations, improvements in diabetes, dyslipidemia, hypertension and obstructive sleep apnea also occur 13.

Postural control assessments are performed by observing body oscillations during orthostatism. The technique used to measure variables related to the body oscillations is called posturography, which is the gold standard for quantitative assessments. The variable most studied in static posturography is the center of pressure (CP), which is the application point resulting from vertical forces that are acting on the supporting surface. The measurement tool most used is the force platform 14. This technique evaluates the sensorial information and motor responses related to postural balance maintenance in an integrated form 15. The test most used to assess flexibility is the sit and reach test 16. These tests are both highly reliable even in obese subjects 17,18. These tests are also quantitative and provide reproducible results for assessing the outcomes and follow-up results of intervention programs 19.

The rapid weight loss caused by the bariatric operation affects postural control and body image. Rapid weight loss also causes changes that are not completely accepted by the patient due to the speed of the process. The lack of recognition of the new body may be one of the factors that creates difficulty in the incorporation of a new lifestyle, which is mainly related to functionality and physical activity practices 20.

## METHODS

In total, 16 patients (both sexes, aged 21 to 60 years old) who received care at the Instituto Central do Hospital das Clínicas da Faculdade de Medicina da Universidade de São Paulo (HCFMUSP), Cirurgia Bariátrica were enrolled.

All volunteers signed the Informed Consent Form (ICF). The research was approved by CAPPesq of HCFMUSP (# 0527/09).

The inclusion criteria included signing the ICF; age between 20 and 60 years old; maximum BMI of 55 before surgery; cognitive level capable of understanding the assessment procedures; ambulatory; absence of chronic or acute musculoskeletal system diseases or disorders from any cause; absence of surgery to the musculoskeletal system; absence of disease or neurological sequelae; absence of untreated or uncontrolled chronic lung and cardiovascular diseases from any cause; absence of dysmetria of the lower limbs of two centimeters or more; and eligibility for bariatric operation.

The sample consisted of 16 subjects (13 (81.2%) female and 3 (18.8%) male) who underwent bariatric surgery. The mean age of the participants was 46.4±10.4 (21-60) years. All assessments were performed before the bariatric operation and at the 6 and 12 month follow-ups.

An initial clinical assessment was performed to collect the following anthropometric data: weight (Kg), height (m) and BMI calculation.

Next, the volunteers underwent a balance assessment on the portable force platform (AccuSwayPlus model, trademark Advanced Mechanical Technology Inc., AMTI, Watertown, MA, USA). The following measurements were performed: CP localization; force measurements (F) and moments (M) in the X (XSD – mediolateral directions), Y (YSD – anteroposterior direction) and Z axes (vertical direction).

After the posturography test, the measurement of flexibility was made using the Sit and Reach test.

### The following variables were assessed:

- Body mass index (BMI) – expressed in kilograms per meter squared (kg/m^2^);
- Flexibility – expressed in centimeters (cm);
- Abdominal perimetry - values used to monitor the flexibility progress with weight loss; expressed in centimeters (cm);
- Average amplitude of displacement from CP in the mediolateral (XSD) and anteroposterior (YSD) planes; measured by the mean square root of displacement from CP in the two directions; expressed in centimeters (cm);
- Elliptical area of 95% displacement from CP with open eyes expressed in centimeters (cm^2^);
- Resultant average speed (VAvg) - average speed of total displacement from CP in all directions divided by collection time; expressed in centimeters per second (cm/s).

#### Statistical Analyses

To evaluate the changes in BMI, flexibility, perimetry, XSD, YSD and displacement area over time, an analysis of variance with repeated measurements was performed. The assumptions of circularity of variances and the covariance matrix were verified through the Mauchly test. When this assumption was not satisfactory, the correction was performed by the Huynh-Feldt test 21. The assumption of normality was assessed through the construction of a normal probability plot of residuals.

In the VAvg analysis, the non-parametric Friedman method was utilized. Initially, the technique of variance analysis with repeated measurements was utilized. However, according to the residual distribution analysis, deviations from the normal distribution required the use of a non-parametric technique.

Correlations between the BMI variation at 6 and 12 months after surgery and flexibility and balance were assessed using Spearman's correlation coefficient 22.

Significance was determined to be 0.05.

The analyses were performed with the aid of Minitab (version 16) and SPSS (version 18) software.

### RESULTS

At 6 months following surgery, the BMI decreased and then stabilized by 12 months. The analysis of variance showed that the BMI values at the three time points measured were not equal (*p*<0.001). According to Tukey's method, the initial BMI (time point 0) was higher than the 6 month (*p*<0.001) and 12 month (*p*<0.001) BMI values following surgery. The 6 and 12 month BMI values were not significantly different (*p*=0.330).

The descriptive statistics indicated no differences regarding displacement in the mediolateral plane (XSD) at the three time points. The analysis of variance indicated no significant differences between the average XSD values at any of the three time points (*p*=0.158).

The descriptive statistics indicated no differences regarding displacement in the anteroposterior plane (YSD) at the three time points. The analysis of variance indicated no significant differences between the averages YSD values at any of the 3 time points (*p*=0.348).

The descriptive statistics indicated no changes in the displacement area values at the three assessments. The analysis of variance indicated no significant differences between the average area values at any of the three time points (*p*=0.219).

The median average speeds of total displacement from CP (VAvg) at 6 and 12 months after the operation were lower than the initial time point. According to the Friedman test, the distributions of VAvg were unequal at the three time points (*p*=0.047). The VAvg values at 6 months after the operation were lower than the initial values (*p*=0.022). The distributions at 6 and 12 months after the procedure did not differ (*p*=0.724). The distributions at baseline and 12 months after the operation did not differ (*p*=0.052).

Flexibility increased over time. According to the analysis of variance, the flexibility differed between the three assessment times (*p*<0.001). According to Tukey's test, the flexibility values were lower at the initial evaluation (time point 0) compared to the values obtained at 6 (*p*=0.031) and 12 months (*p*<0.001) after the operation. The flexibility values at 6 months were lower than the values obtained at 12 months (*p*=0.003) after the operation.

All results are shown in Table 1.

### DISCUSSION

According to our findings, obesity is a difficult-to-treat, multifactorial disease that causes and worsens cardiovascular, hormonal and musculoskeletal alterations. Additionally, the high prevalence of obesity is an epidemic, and bariatric surgery is an alternative treatment. The effects of rapid body weight loss are not completely known, especially regarding the musculoskeletal system and motor control. Musculoskeletal complications after bariatric operations can be associated with nutritional deficiencies but also to the absence of orientation and a lack of adherence to healthy lifestyle choices, such as a healthy diet and physical activity.

The results of this study are relatively novel. No previous publications have reported balance and flexibility changes of obese subjects before and after bariatric procedures.

BMI values decrease after 6 months and was stabilized by the 12 month assessment.

According to the posturography performed with the force platform, the rapid weight loss did not interfere with the displacement from CP in the mediolateral and anteroposterior directions or the displacement speed. These variables, therefore, are possibly more dependent on the integration of the entire neuromuscular system of balance maintenance. Even with extreme weight loss and BMI reductions, the distance and area of displacement from CP do not significantly change. This effect is possibly due to the long adaptation period of the subject to being overweight. This adaptation is necessary to maintain postural balance and maintain the CP inside the supporting area. This adaptation is also necessary to maintain orthostatic position, gait and to avoid falls. These parameters may be more closely associated with learning than body mass.

The rapid weight loss did not change the size of the supporting basis or the displacement distances from CP. This result may be due to other factors; perhaps the proprioceptive system may have not had time to adapt to the new body weight. The lowered body weight and higher proportional lean mass may modify the CP displacement parameters. We can infer, according to these results, that the spatial parameter modifications in static balance are more neuromuscular than mechanic, and more time may be required to adapt and observe improvements. In the study by Alonso et al. 23, a population study without restrictions on body weight or BMI, these metrics did not influence the static postural balance parameters obtained via the force platform. However, based on 59 male subjects assessed on the force platform, Hue et al. 24 stated that an increase in body weight correlated with higher static balance instability.

The displacement speed from CP, which decreased after 6 months and increased again after 12 months, corroborates the above data. The response speed changes and corrections to maintain static balance occur faster but, over time, the values return to baseline. The correction speed is the most important and volatile parameter affecting the maintenance of CP inside the supporting base. The displacement from CP would be more automatic and structured if it was a neuromuscular response and, therefore, less likely to changes.

Flexibility increased over time. The flexibility increases observed at the two reassessments may be related to the decreased body measurements, body mass and BMI. Corporal fat decreases the level of flexibility and worsens motor performance 25. Therefore, increased flexibility is an important factor affecting the functional improvement of surgical patients. Flexibility also increases the efficiency of movements contributing to the prevention of lesions and improves corporal conscience, which is impaired in patients following bariatric operations who do not recognize their new body. The improvements in flexibility improve motor control, facilitate the execution of movements and aid in recognizing the new body image 26,27,28.

The flexibility improvements were not reflected as static balance gains. The adaptation of the patient to the new body mass and the lack of specific training may explain the lack of improvement in balance assessed by displacement from CP. These data agree with the findings of Bankoff et al. Heavier bodies are harder to unbalance, but weight does not affect the displacement speed from CP 29. Ferreira 30 stated that the body mass interferes with the position (height) from CP 30. Gain or loss of body mass affects the height from CP and balance and also depends on the mass distribution of the body.

In obese subjects who underwent bariatric surgery, the static postural balance did not change when the displacement from CP was assessed 6 and 12 months after the operation. There is greater demand on the proprioceptive system (motor control) to maintain balance, as indicated by the increased speed of postural adjustment responses at 6 months. Weight loss promotes increased flexibility, although there is no correlation between the improvements in flexibility and balance.

### AUTHOR CONTRIBUTIONS

Benetti FA, main author, conceived the study, collected the data, participated in the analysis of the samples, drafted the manuscript, and participated in the statistical analyses. Bacha IL, co-author, conceived the study and participated in study design and coordination and manuscript drafting. D'Andréa Greve JM and Garrido Jr AB, co-authors and supervisors, participated in data analyses and interpretation.

## Figures and Tables

**Table 1:** Average and standard deviation of Body Mass Index, flexibility, X standard deviation, Y standard deviation, 95% area under the curve and Vavg at the time of surgery (time 0), 6 and 12 months after surgery.

Variables	Time after surgery (months)		
		**0**	**6**	**12**
			**Average (SD)**			
BMI (Kg/m2)	44.6 (4.5)	32.6 (2.7)	31 (3.0)
Flexibility (cm)	20.2 (9.1)	24.8 (6.7)	29.8 (8.1)
X SD (cm)	0.340 (0.248)	0.325 (0.149)	0.269 (0.109)
Y SD (cm)	0.525 (0.288)	0.447 (0.125)	0.491 (0.097)
Area 95%	2.863 (2.335)	2.668 (1.749)	2.312 (1.301)
Vavg (cm/sec)	0.937 (0.385)	0.930 (0.586)	0.876 (0.302)

SD: standard deviation; BMI: Body Mass Index; X SD: Average amplitude of pressure center displacement in the mediolateral plane; Y SD: Average amplitude of the center of pressure displacement in the anteroposterior plane; Area 95%: Elliptical area of 95%; VAvg: Average Resultant Speed.
